# Correction: Expansion microscopy reveals subdomains in *C. elegans* germ granules

**DOI:** 10.26508/lsa.202302405

**Published:** 2023-10-09

**Authors:** Kin M Suen, Thomas MD Sheard, Chi-Chuan Lin, Dovile Milonaityte, Izzy Jayasinghe, John E Ladbury

**Affiliations:** 1 School of Molecular and Cellular Biology, University of Leeds, Leeds, UK; 2 School of Biosciences, University of Sheffield, Sheffield, UK

## Abstract

Correction to Materials and Methods.

Referring to the following published article:

Article: Suen KM, Sheard TMD, Lin C-C, Milonaityte D, Jayasinghe I, Ladbury JE (2023 Feb 7) Expansion microscopy reveals subdomains in *C. elegans* germ granules. Life Sci Alliance 6(4): e202201650. doi: 10.26508/lsa.202201650. PMID: 36750365.

Correction to the Materials and Methods: Gel expansion.

“Excess liquid was removed as much as possible without drying the germlines before the addition of monomer solution (8.6 mg/ml sodium acrylate (461652; Fluorochem), 2.5 mg/ml acrylamide, 0.15 mg/ml N,N′-methylenebisacrylamide (M7279; Merck), and 11.7 mg/ml NaCl in 1× PBS).”

Should read the following:

“Excess liquid was removed as much as possible without drying the germlines before the addition of monomer solution (86 mg/ml sodium acrylate (461652; Fluorochem), 25 mg/ml acrylamide, 1.5 mg/ml N,N′-methylenebisacrylamide (M7279; Merck), and 117 mg/ml NaCl in 1× PBS).”

## Supplementary Material

Reviewer comments

