# Supplementary material for: Correction: Expansion microscopy reveals subdomains in C. elegans germ granules
Source: Life Sci Alliance. 2023 Oct 9;7(1):e202302405. doi: 10.26508/lsa.202302405 (PMC10561762; doi:10.26508/lsa.202302405)
Supplement: Reviewer comments [file LSA-2023-02405_review_history.pdf]

Life Science Alliance

Correction: Expansion microscopy reveals subdomains in C. elegans germ granules

John Ladbury, Kin Suen, Thomas Sheard, Chi-Chuan Lin, Dovilė Milonaitytė, and Izzy Jayasinghe
DOI: https://doi.org/10.26508/lsa.202302405

Corresponding author(s): John Ladbury, University of Leeds and Kin Suen, University of Leeds

**Review Timeline:**	Submission Date:	2023-09-28
	Accepted:	2023-10-02

Scientific Editor: Eric Sawey, PhD

Transaction Report:

(Note: With the exception of the correction of typographical or spelling errors that could be a source of ambiguity, letters and reports are not edited. The original formatting of letters and referee reports may not be reflected in this compilation.)

October 2, 2023

RE: Life Science Alliance Manuscript #LSA-2023-02405

Prof. John E Ladbury
University of Leeds
Cellular & Molecular Biology
Room 7.08, Miall Building
Leeds, North Yorkshire LS2 9JT
United Kingdom

Dear Dr. Ladbury,

Thank you for submitting your Correction entitled "Correction: Expansion microscopy reveals subdomains in C. elegans germ granules".

The final published version of your Correction will be deposited by us to PubMed Central upon online publication.

IMPORTANT: If you will be unreachable at any time, please provide us with the email address of an alternate author. Failure to respond to routine queries may lead to unavoidable delays in publication.

Scheduling details will be available from our production department.

You can contact the journal office with any questions, contact@life-science-alliance.org

Sincerely,

Eric Sawey, PhD
Executive Editor
Life Science Alliance
http://www.lsajournal.org

October 2, 2023

RE: Life Science Alliance Manuscript #LSA-2023-02405

Prof. John E Ladbury
University of Leeds
Cellular & Molecular Biology
Room 7.08, Miall Building
Leeds, North Yorkshire LS2 9JT
United Kingdom

Dear Dr. Ladbury,

Thank you for submitting your Correction entitled "Correction: Expansion microscopy reveals subdomains in C. elegans germ granules".

The final published version of your Correction will be deposited by us to PubMed Central upon online publication.

IMPORTANT: If you will be unreachable at any time, please provide us with the email address of an alternate author. Failure to respond to routine queries may lead to unavoidable delays in publication.

Scheduling details will be available from our production department.

You can contact the journal office with any questions, contact@life-science-alliance.org

Sincerely,

Eric Sawey, PhD
Executive Editor
Life Science Alliance
http://www.lsajournal.org
